# Behavioral effects of zonisamide on L-DOPA-induced dyskinesia in Parkinson's disease model mice

**DOI:** 10.3389/fnagi.2023.1221341

**Published:** 2023-06-27

**Authors:** Hiromi Sano, Atsushi Nambu

**Affiliations:** ^1^Division of Behavioral Neuropharmacology, International Center for Brain Science, Fujita Health University, Toyoake, Japan; ^2^Division of System Neurophysiology, National Institute for Physiological Sciences, Okazaki, Japan; ^3^Department of Physiological Sciences, SOKENDAI (The Graduate University for Advanced Studies), Okazaki, Japan

**Keywords:** Parkinson's disease, L-DOPA-induced dyskinesia, basal ganglia, zonisamide, L-DOPA

## Abstract

Zonisamide (ZNS; 1,2-benzisoxazole-3-methanesulfonamide) was initially developed and is commonly used as an anticonvulsant drug. However, it has also shown its beneficial effects on Parkinson's disease (PD), a progressive neurodegenerative disorder caused by the loss of dopaminergic neurons in the midbrain. Recent clinical studies have suggested that ZNS can also have beneficial effects on L-DOPA-induced dyskinesia (LID), which is a major side effect of long-term L-DOPA treatments for PD. In the present study, we examined the behavioral effects of ZNS on LID in PD model mice. Acute ZNS treatment did not have any observable behavioral effects on LID. Contrastingly, chronic ZNS treatment with L-DOPA delayed the peak of LID and reduced the severity of LID before the peak but increased the duration of LID in a dose-dependent manner of ZNS compared to PD model mice treated with L-DOPA alone. Thus, ZNS appears to have both beneficial and adverse effects on LID.

## Introduction

Parkinson's disease (PD) is a neurodegenerative disorder caused by the loss of nigrostriatal dopaminergic neurons in the substantia nigra pars compacta (SNc), leading to motor and non-motor symptoms, including akinesia, bradykinesia, resting tremor, rigidity, cognitive dysfunctions, depression, and insomnia (Fahn, 2003; Blesa et al., 2022). Supplementation therapy with l-DOPA can effectively control symptoms in the early stage of PD (a well-controlled “honeymoon” period) (Fahn, 2003), but long-term administration can induce wearing-off, a reduction of the effective time, and involuntary movements known as l-DOPA-induced dyskinesia (LID). Once LID is established, it is difficult to relieve involuntary movements without compromising antiparkinsonian effects (Jenner, 2008). The reduction and prevention of LID are major issues in PD drug therapy.

Zonisamide (ZNS; 1,2-benzisoxazole-3-methanesulfonamide) was initially approved for epilepsy treatment in Japan in 1989 and later approved worldwide (Kwan et al., 2015). It was also serendipitously discovered to have beneficial effects on the symptoms of PD (Murata et al., 2001, 2007; Murata, 2004) at a lower dose (25–50 mg/day) than the therapeutic doses against epilepsy. When ZNS was administered to a patient with PD who incidentally had convulsive attacks, the attacks disappeared, and the symptoms of PD also improved. Several clinical trials have explored the effects of ZNS on the motor symptoms of PD, and in 2009, it was approved as an adjunctive treatment for patients with PD who responded insufficiently to l-DOPA treatment in Japan. ZNS significantly reduces the duration of wearing off without worsening dyskinesia (Murata et al., 2015). Although the mechanism of ZNS in the symptoms of PD has not been fully elucidated, it has been suggested to have multiple mechanisms of action, including the inhibition of monoamine oxidase (Sonsalla et al., 2010; Binda et al., 2011), blockade of T-type calcium channels (Suzuki et al., 1992; Kito et al., 1996), modulation of l-DOPA-dopamine metabolism (Nishijima et al., 2018), and neuroprotection (Asanuma et al., 2010; Sano et al., 2015).

In our previous study, we investigated the behavioral and neurophysiological effects of ZNS on LID in PD model mice (Sano and Nambu, 2019). The chronic ZNS injection (100 mg/kg) in combination with l-DOPA increased the duration and severity of LID and altered neuronal activity in the output nuclei of the basal ganglia, i.e., the substantia nigra pars reticulata (SNr). This resulted in a decreased spontaneous firing rate, increased cortically evoked inhibition, and decreased cortically evoked late excitation, which may explain the mechanism of enhanced LID (Sano and Nambu, 2019; Dwi Wahyu et al., 2021). In this study, we aimed to evaluate the behavioral effects of ZNS on LID in more detail by injecting l-DOPA or a combination of l-DOPA and ZNS at various doses to induce LID in PD model mice. We observed abnormal involuntary movements (AIMs) after l-DOPA with or without ZNS injection.

## Materials and methods

### Animals

Male ICR mice were obtained from Japan SLC. All the mice used in the experiments were more than 8 weeks old. The mice were maintained on a 12-h light and dark cycle with *ad libitum* access to food and water. All experimental procedures were approved by the Institutional Animal Care and Use Committee of the National Institutes of Natural Sciences.

### Experimental design

**Figure 1:**
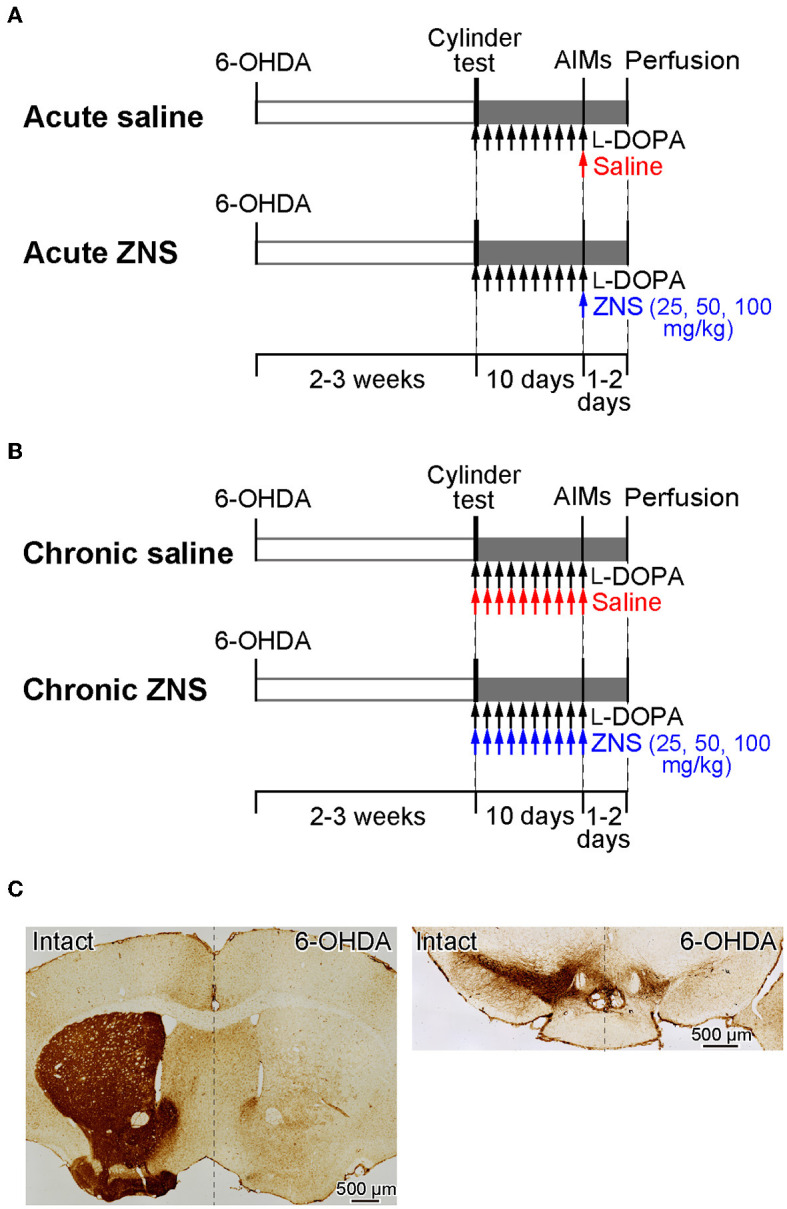
A schematic representation of the experimental design. **(A, B)** Timelines of Experiments 1 and 2. Mice received 6-OHDA infusions into the right medial forebrain bundle. The cylinder test was performed to evaluate motor deficits 2 to 3 weeks after injection. **(A)** Experiment 1 was performed to evaluate the acute effects of ZNS on LID. After the cylinder test, mice were injected with l-DOPA for 9 consecutive days (20 mg/kg; black arrows), and behavioral tests for AIMs were conducted on the 10th day after l-DOPA with saline (red arrow) or ZNS (25, 50, and 100 mg/kg; blue arrow) injections. **(B)** Experiment 2 was performed to evaluate the chronic effects of ZNS on LID. After the cylinder test, mice were injected with l-DOPA (20 mg/kg; black arrows) with saline (red arrows) or ZNS (25, 50, and 100 mg/kg; blue arrows) for 9 consecutive days, and AIMs were observed on the 10th day after l-DOPA with saline or ZNS injection. **(C)** Dopaminergic nerve terminals in the striatum (left panel) and dopaminergic neurons in the substantia nigra pars compacta (right panel) on the intact and 6-OHDA-lesioned sides were visualized with tyrosine hydroxylase immunohistochemistry. 6-OHDA, 6-hydroxydopamine; AIMs, abnormal involuntary movements; LID, l-DOPA-induced dyskinesia; ZNS, zonisamide.

To investigate the effects of acute (Experiment 1, Figure 1A) and chronic (Experiment 2, Figure 1B) ZNS injection on LID, we performed the following experiments. First, mice received a 6-hydroxydopamine (6-OHDA) injection into the right medial forebrain bundle (MFB) to eliminate dopaminergic neurons. After 2 to 3 weeks, motor deficits were evaluated using the cylinder test. In Experiment 1, after injecting l-DOPA for 9 consecutive days, we observed dyskinetic behavior after l-DOPA with saline or ZNS injections (Figure 1A). In Experiment 2, after injecting l-DOPA with saline or ZNS for 9 consecutive days, we observed dyskinetic behavior after l-DOPA with saline or ZNS injections (Figure 1B). After 1 or 2 days of the behavioral test, the mice were perfused transcardially with formalin, and histological analysis was performed to evaluate the degeneration of dopaminergic neurons in the SNc.

### 6-OHDA lesion

6-OHDA was infused as described in previous studies (Sano and Nambu, 2019; Dwi Wahyu et al., 2021). Briefly, desipramine hydrochloride (25 mg/kg, *i.p*.; Sigma-Aldrich) was injected into mice 30 min before the 6-OHDA infusion. Mice were anesthetized with isoflurane (1.0–1.5%) and fixed in a stereotaxic apparatus (SR-6M-HT, Narishige). The skin of the head was incised, and a small hole was made on the right side of the skull. We injected 6-OHDA-hydrobromide (4 mg/ml; Sigma-Aldrich) that was dissolved in saline containing 0.02% ascorbic acid into the right medial forebrain bundle at the coordinates 1.0-mm posterior, 1.4-mm lateral, and 4.8-mm ventral from the bregma, using a glass micropipette and a micro infusion pump (1 μl at 0.2 μl/min; Micro4, WPI).

### Cylinder test

To evaluate motor deficits induced by a unilateral lesion of the nigrostriatal dopaminergic neurons, a cylinder test was performed 2 to 3 weeks after the 6-OHDA infusion, as described in previous studies (Figures 1A, B) (Tillerson et al., 2001; Sano and Nambu, 2019; Dwi Wahyu et al., 2021). Each mouse was placed in a clear acrylic cylinder (10 cm in diameter and 20 cm in height) and was observed for 5 min without habituation to the cylinder. The number of contacts with the right or left forelimb was counted, and the limb-use asymmetry score was calculated as the number of wall contacts with the left forelimb (the forelimb contralateral to the lesion side) as a percentage of the total wall contacts. The limb-use asymmetry score was expected to be ≈30%. Nearly 90% of dopaminergic neurons were lesioned in these mice, based on our previous experiments (Sano and Nambu, 2019; Dwi Wahyu et al., 2021). If mice showed a limb-use asymmetry score of >33%, 6-OHDA was injected again after waiting for the recovery of body weight to the level before the first injection (37.3% of mice), so the limb-use asymmetry score was ≈30%. These mice with two injections did not show apparent differences from mice with one injection.

### L-DOPA and ZNS treatment

Daily l-DOPA treatment was initiated after the cylinder test. The 6-OHDA-lesioned mice were injected with l-DOPA (Experiment 1, Figure 1A) or l-DOPA with saline or ZNS (Experiment 2, Figure 1B) once a day for 9 consecutive days. l-DOPA was injected (20 mg/kg, *i.p*.; Ohara Pharmaceutical Co., Ltd) in combination with benserazide (12 mg/kg, *i.p*.; Sigma-Aldrich). In Experiment 2, saline (10 ml/kg, *i.p*.) or different doses of ZNS (25, 50, and 100 mg/kg, *i.p*.; provided by Sumitomo Pharma Co., Ltd.) were injected 10 min after l-DOPA treatment.

### Behavioral test

On day 10, after injecting l-DOPA with or without ZNS treatments for 9 consecutive days (Experiments 1 and 2, respectively), saline (10 ml/kg, *i.p*.) or different doses of ZNS (25, 50, and 100 mg/kg, *i.p*.) were injected 10 min after l-DOPA (20 mg/kg, *i.p*.) treatment. The mice were then placed in separate cages, and dyskinetic behaviors were observed every 20 min for 1 min over a 180-min period (Figures 1A, B). Dyskinetic behavior was scored based on the previously described AIMs scale (Sano and Nambu, 2019; Dwi Wahyu et al., 2021), which classifies AIMs into four subtypes: locomotive (increased locomotion with contralateral rotations), axial (contralateral dystonic posture of the neck and upper body toward the side contralateral to the lesion), limb (jerky and fluttering movements of the limb contralateral to the side of the lesion), and orolingual AIMs (vacuous jaw movements and tongue protrusions). Each of these subtypes was scored on a severity scale ranging from 0 to 4 (0, absent; 1, occasional; 2, frequent; 3, continuous; 4, continuous, and not interruptible by external stimuli). Furthermore, we calculated the total AIMs score by adding up the four AIM subtypes.

### Histology

After 1 to 2 days of the behavioral tests, the mice were deeply anesthetized with sodium thiopental (100 mg/kg, *i.p*.) and were perfused transcardially with 0.01M phosphate-buffered saline (PBS) followed by 10% formalin in 0.01M PBS. The brains were removed, postfixed in 10% formalin at 4°C overnight, cryoprotected in 15% sucrose in 0.01M PBS at 4°C, and then in 30% sucrose in 0.01M PBS at 4°C. These brains were subsequently frozen and sectioned coronally into 40-μm sections. The free-floating sections were incubated with antibodies against tyrosine hydroxylase (TH; 1:500; Millipore) and then visualized with biotinylated secondary antibodies and the ABC method (Sano et al., 2015).

### Statistical analysis

l-DOPA-induced AIMs were compared among the four groups (saline and different doses of ZNS) using a two-way analysis of variance (ANOVA) with repeated measures and Tukey's *post hoc* test. Statistical analyses were performed using Prism7 software (GraphPad Software Inc.), and a *p*-value of < 0.05 was considered to be statistically significant.

## Results

TH-positive terminals in the striatum and TH-positive neurons in the SNc dramatically decreased on the 6-OHDA injection side, as shown in Figure 1C. In the cylinder test, mice treated with 6-OHDA showed a significant reduction in the usage of the forelimb contralateral to the lesion, and the limb-use asymmetry scores were decreased to 28.91 ± 6.09 % (mean ± S.D.), which was similar across the four groups in Experiments 1 and 2. These results confirmed that the dopaminergic neurons in the SNc were successfully lesioned and that the mice exhibited the symptoms of PD.

### Effect of acute ZNS treatment on AIMs, experiment 1

**Figure 2:**
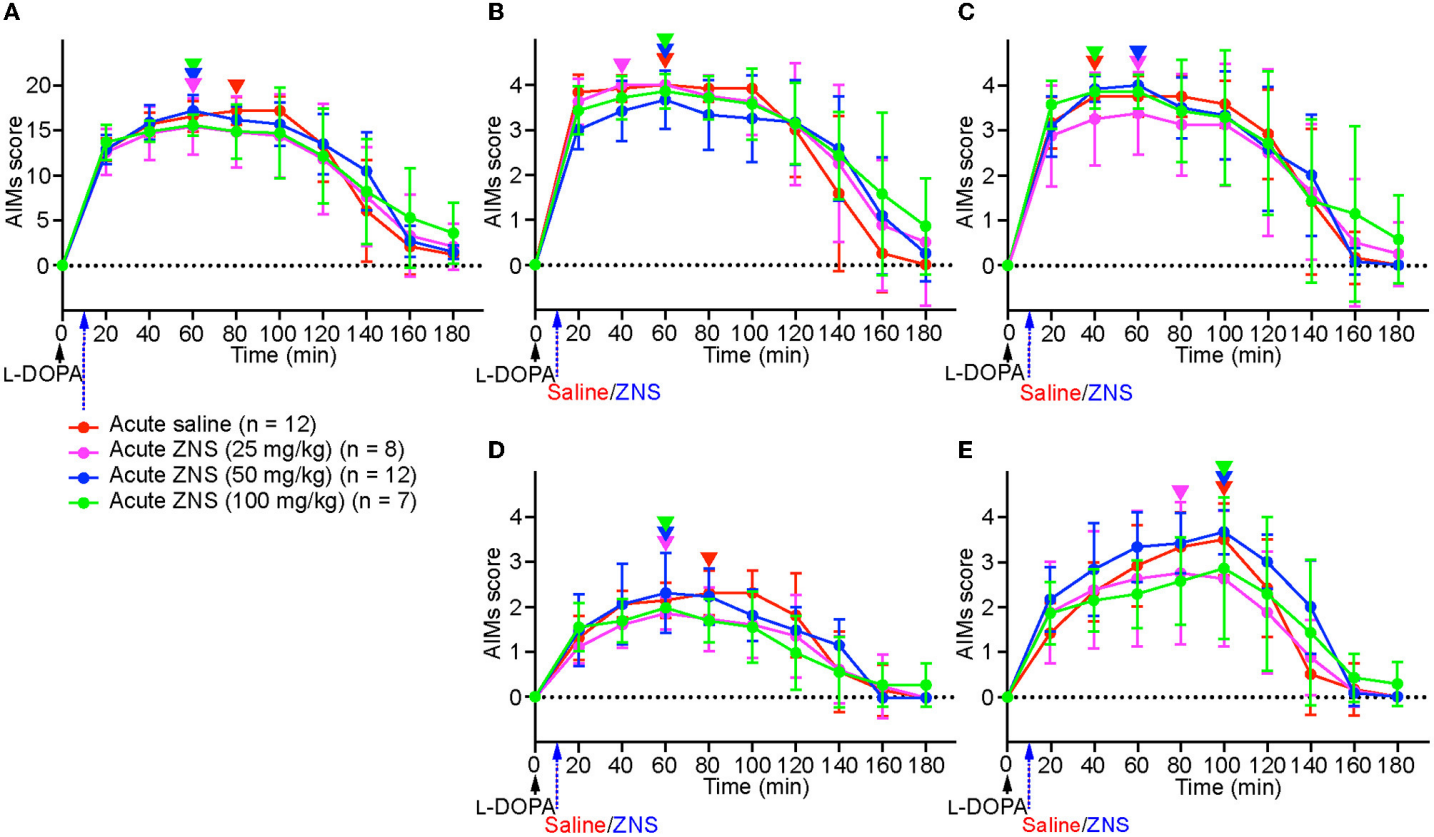
Acute ZNS treatment does not affect AIMs scores. Total **(A)**, locomotive **(B)**, axial **(C)**, limb **(D)**, and orolingual **(E)** AIMs were scored for 180 min at every 20 min after injecting l-DOPA (20 mg/kg; black arrow) at time 0 min with saline (red) or ZNS (25 mg/kg, magenta; 50 mg/kg, blue; 100 mg/kg, green) at 10 min. *n*, number of animals in each group. Data are expressed as mean ± SD. Peaks of AIMs with saline or ZNS are indicated by inverted triangles with corresponding colors. AIMs, abnormal involuntary movements; ZNS, zonisamide.

On day 10, after injecting l-DOPA for 9 consecutive days, saline or ZNS (25, 50, and 100 mg/kg) was injected 10 min after l-DOPA treatment (Figure 1A), and AIMs were observed in different body parts and were scored for 1 min at every 20 min after l-DOPA treatment (Figure 2). We hardly observed tremors in each group. We compared the total AIMs scores between the acute saline and ZNS (25, 50, and 100 mg/kg) treatment groups. There was no statistically significant difference between all groups [Figure 2A; two-way repeated measures ANOVA: time, *F*_(8, 280)_ = 169.4, *p* < 0.01; treatment, *F*_(3, 35)_ = 0.36, *p* = 0.78; interaction, *F*_(24, 280)_ = 1.83, *p* < 0.05]. There was also no significant difference between all groups in the locomotive [Figure 2B; two-way repeated measures ANOVA: time, *F*_(8, 280)_ = 99.68, *p* < 0.01; treatment, *F*_(3, 35)_ = 0.65, *p* = 0.59; interaction, *F*_(24, 280)_ = 1.81, *p* < 0.05], axial [Figure 2C; two-way repeated measures ANOVA: time, *F*_(8, 280)_ = 114.1, *p* < 0.01; treatment, *F*_(3, 35)_ = 0.38, *p* = 0.77; interaction, *F*_(24, 280)_ = 1.08, *p* = 0.37], limb [Figure 2D; two-way repeated measures ANOVA: time, *F*_(8, 280)_ = 82.34, *p* < 0.01; treatment, *F*_(3, 35)_ = 1.64, *p* = 0.20; interaction, *F*_(24, 280)_ = 1.80, *p* < 0.05], and orolingual [Figure 2E; two-way repeated measures ANOVA: time, *F*_(8, 280)_ = 115.3, *p* < 0.01; treatment, *F*_(3, 35)_ = 1.64, *p* = 0.20; interaction, *F*_(24, 280)_ = 2.51, *p* < 0.01] AIMs scores. We compared the peaks of AIMs in the acute saline and ZNS (25, 50, and 100 mg/kg) treatment groups (inverted triangles in Figure 2). Peaks of the total, locomotive, axial, limb, and orolingual AIMs scores were distributed between 40 and 100 min. These results suggest that acute ZNS injections did not affect l-DOPA-induced AIMs in PD model mice.

### Effect of chronic ZNS treatment on AIMs, experiment 2

**Figure 3:**
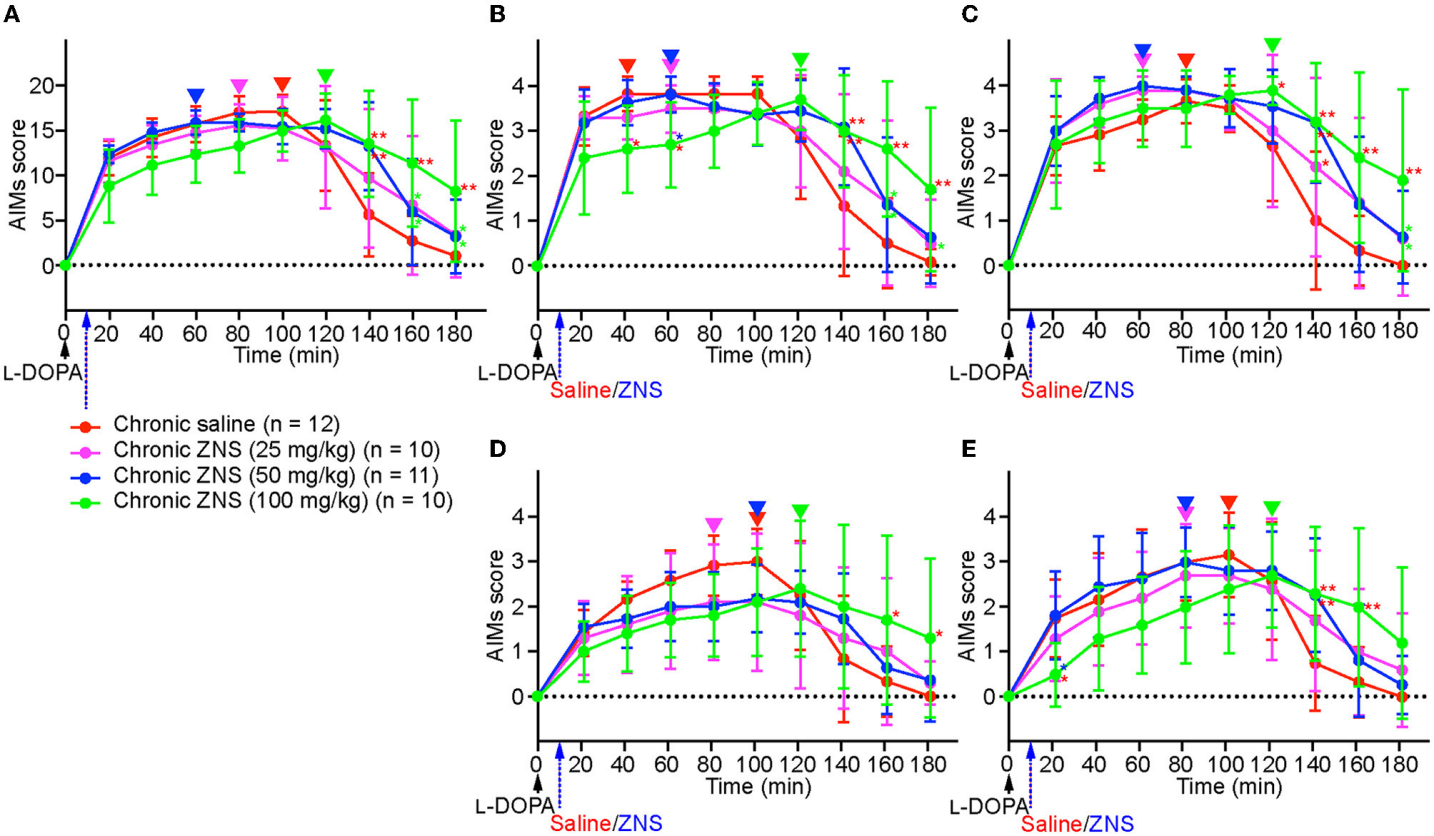
Chronic ZNS treatment delayed the peak of AIMs and decreased AIM scores before their peak but increased their duration. Total **(A)**, locomotive **(B)**, axial **(C)**, limb **(D)**, and orolingual **(E)** AIMs were scored for 180 min at every 20 min after injecting l-DOPA (20 mg/kg; black arrow) at time 0 min with saline (red) or ZNS (25 mg/kg, magenta; 50 mg/kg, blue; 100 mg/kg, green) at 10 min. *n*, number of animals in each group. Data are expressed as mean ± SD. *p < 0.05, **p < 0.01 indicates significant differences between different treatments with the corresponding color (two-way repeated measures ANOVA followed by Turkey's *post hoc* test). Peaks of AIMs with saline or ZNS are indicated by inverted triangles with the corresponding colors. AIMs, abnormal involuntary movements; ZNS, zonisamide.

On day 10, after injecting l-DOPA with saline or ZNS (25, 50, and 100 mg/kg) for 9 consecutive days (Figure 1B), AIMs were observed in different body parts and were scored for 1 min at every 20 min after l-DOPA with saline or ZNS treatment (Figure 3). We hardly observed tremor in each group. We compared the total AIMs scores between chronic saline and ZNS (25, 50, and 100 mg/kg) treatments (Figure 3A; two-way repeated measures ANOVA: time, *F*_(8, 312)_ = 57.44, *p* < 0.01; treatment, *F*_(3, 39)_ = 1.12, *p* = 0.35; interaction, *F*_(24, 312)_ = 4.80, *p* < 0.01). Chronic ZNS (50 and 100 mg/kg) treatment showed significantly higher AIMs scores than chronic saline treatment at 140 min (Tukey's *post hoc* test: saline vs. ZNS [50 and 100 mg/kg], *p* < 0.01). Chronic ZNS (100 mg/kg) treatment also resulted in significantly higher AIMs scores than the other three groups at 160 and 180 min [saline vs. ZNS (100 mg/kg), *p* < 0.01; ZNS (25 and 50 mg/kg) vs. ZNS (100 mg/kg), *p* < 0.05].

Next, we compared the AIMs scores for each body part. In locomotive AIMs scores [Figure 3B; two-way repeated measures ANOVA: time, *F*_(8, 312)_ = 49.04, *p* < 0.01; treatment, *F*_(3, 39)_ = 1.01, *p* = 0.40; interaction, *F*_(24, 312)_ = 4.32, *p* < 0.01], chronic ZNS (100 mg/kg) treatment showed lower AIMs scores at 40 min [Tukey's *post hoc* test: saline vs. ZNS (100 mg/kg), *p* < 0.05] and 60 min [saline and ZNS (50 mg/kg) vs. ZNS (100 mg/kg), *p* < 0.05]. However, chronic ZNS (100 mg/kg) treatment showed higher AIMs scores at 140 min [saline vs. ZNS (50 and 100 mg/kg), *p* < 0.01], 160 min [saline vs. ZNS (100 mg/kg), *p* < 0.01; ZNS (25 and 50 mg/kg) vs. ZNS (100 mg/kg), *p* < 0.05], and 180 min [saline vs. ZNS (100 mg/kg), *p* < 0.01; ZNS (25 mg/kg) vs. ZNS (100 mg/kg), *p* < 0.05]. In axial AIMs scores [Figure 3C; two-way repeated measures ANOVA: time, *F*_(8, 312)_ = 50.75, *p* < 0.01; treatment, *F*_(3, 39)_ = 7.02, *p* < 0.01; interaction, *F*_(24, 312)_ = 2.27, *p* < 0.01], chronic ZNS (100 mg/kg) treatment showed higher AIMs scores at 120 min [Tukey's *post hoc* test: saline vs. ZNS (100 mg/kg), *p* < 0.05], 140 min [saline vs. ZNS (25 mg/kg), *p* < 0.05; saline vs. ZNS (50 and 100 mg/kg), *p* < 0.01], 160 min [saline vs. ZNS (100 mg/kg), *p* < 0.01], and 180 min [saline vs. ZNS (100 mg/kg), *p* < 0.01; ZNS (25 and 50 mg/kg) vs. ZNS (100 mg/kg), *p* < 0.05]. In limb AIMs scores [Figure 3D; two-way repeated measures ANOVA: time, *F*_(8, 312)_ = 31.18, *p* < 0.01; treatment, *F*_(3, 39)_ = 0.19, *p* = 0.90; interaction, *F*_(24, 312)_ = 4.03, *p* < 0.01], chronic ZNS (100 mg/kg) treatment showed higher AIMs scores at 160 and 180 min [Tukey's *post hoc* test: saline vs. ZNS (100 mg/kg), *p* < 0.05]. In orolingual AIMs scores [Figure 3E; two-way repeated measures ANOVA: time, *F*_(8, 312)_ = 32.01, *p* < 0.01; treatment, *F*_(3, 39)_ = 0.42, *p* = 0.74; interaction, *F*_(24, 312)_ = 3.70, *p* < 0.01], chronic ZNS (100 mg/kg) treatment showed lower AIMs scores at 20 min [Tukey's *post hoc* test: saline and ZNS (50 mg/kg) vs. ZNS (100 mg/kg), *p* < 0.05]. In contrast, chronic ZNS (100 mg/kg) treatment resulted in higher AIMs scores at 140 min [saline vs. ZNS (50 and 100 mg/kg), *p* < 0.01] and 160 min [saline vs. ZNS (100 mg/kg), *p* < 0.01].

We compared the peaks of AIMs in the chronic saline and ZNS (25, 50, and 100 mg/kg) treatment groups (inverted triangles in Figure 3). Peaks of the total, locomotive, axial, limb, and orolingual AIMs of chronic saline and ZNS (25 and 50 mg/kg) treatments were distributed between 40 and 100 min; however, those of the chronic ZNS (100 mg/kg) treatments were at 120 min.

These results indicate that chronic ZNS injection with l-DOPA in PD model mice decreased locomotive and orolingual AIMs scores before the peak and delayed the peak of the total, locomotive, axial, limb, and orolingual AIMs but increased the duration of the total, locomotive, axial, limb, and orolingual AIMs in a ZNS dose-dependent manner compared with PD model mice that underwent l-DOPA and chronic saline treatments.

## Discussion

The present study yielded the following results: (1) acute ZNS injection had no effects on l-DOPA-induced AIMs and (2) chronic ZNS injection delayed the peak of all AIMs and decreased some AIMs scores before the peak but increased the duration of all AIMs in a ZNS dose-dependent manner.

On day 10 after injecting l-DOPA for 9 consecutive days, acute ZNS injection at all doses (25–100 mg/kg) did not enhance nor prolong AIMs in PD model mice (Figure 2). This result suggests that acute ZNS injection does not improve or worsen the symptoms of LID and is consistent with a previous study on PD model rats that received l-DOPA (6 mg/kg) with ZNS (50 mg/kg) (Nishijima et al., 2018). This study suggested that there was no significant difference between the l-DOPA-only and l-DOPA with ZNS groups in AIMs on day 1. A single ZNS administration may not affect LID although acute ZNS administration (300 mg) improves symptoms in PD patients (Murata et al., 2001).

In contrast, in the present study, chronic ZNS injection (100 mg/kg) with l-DOPA (20 mg/kg) delayed the peak of AIMs and decreased AIMs scores before the peak but prolonged the duration of AIMs in a dose-dependent manner (Figure 3). Previous reports have shown inconsistent effects of ZNS on AIMs in PD model rats. AIMs were observed on the 15th day of l-DOPA (6 mg/kg) with ZNS (50 mg/kg) treatment after 14 consecutive injections once a day (Nishijima et al., 2018). The AIMs of l-DOPA in the ZNS group were enhanced and prolonged, and the peaked AIMs score worsened slightly. Recent reports have shown that ZNS ameliorates LID in PD model rats (Oki et al., 2017; Tohge et al., 2021), which included l-DOPA (12 mg/kg) and ZNS (26 mg/kg) injections twice a day for 2 weeks and AIMs evaluation. The AIMs of the l-DOPA with ZNS-injected group were statistically lower than those of the l-DOPA-only injected group 140 min after injection (Oki et al., 2017). The effect of ZNS might be increased by the twice-daily injection of ZNS compared to the once-daily injection. The daily dosage of l-DOPA might explain the different effects of ZNS on LID: 6 mg/kg/day in Nishijima et al. (2018) vs. 24 mg/kg/day in Oki et al. (2017) and Tohge et al. (2021) and 20 mg/kg/day in the present study.

Although the precise mechanism, by which ZNS affects LID is not fully elucidated, previous studies have demonstrated that l-DOPA with ZNS treatment normalizes the expression of several key molecules in the striatum, such as adenosine A_2A_, endocannabinoid CB_1_, and 5-HT_1B_ receptors, which are upregulated in 6-OHDA-treated rats with LID (Oki et al., 2017; Tohge et al., 2021). Additionally, the recording of neuronal activity in the SNr, one of the output nuclei of the basal ganglia, in PD model mice treated with l-DOPA and ZNS injections has revealed changes in response to motor cortical stimulation (Sano and Nambu, 2019). In normal mice, this stimulation induces a triphasic response composed of early excitation, inhibition, and late excitation in the SNr (Sano et al., 2013; Chiken et al., 2015; Sano and Nambu, 2019; Dwi Wahyu et al., 2021). However, in LID model mice, longer inhibition and reduced late excitation were observed in the SNr (Dwi Wahyu et al., 2021). l-DOPA with ZNS treatment further enhances these changes, which are mediated by the cortico-striato-SNr *direct* and cortico-striato-external pallido-subthalamo-SNr *indirect* pathways, respectively (Tachibana et al., 2008; Sano et al., 2013; Chiken et al., 2015). Signals through the *direct* pathway inhibit the SNr activity and release appropriate movements, while those through the *indirect* pathway increase the SNr activity and induce clear termination of movements (Mink and Thach, 1993; Nambu et al., 2002). Therefore, l-DOPA with ZNS treatment enhances LID responses in the SNr and likely causes prolonged LID. In addition, ZNS inhibits low-voltage-activated Ca^2+^ currents in subthalamic neurons (Yang et al., 2014) and may reduce activity through the *indirect* pathway and cortically evoked late excitation in the SNr, enhancing LID. In the present study, the effects of ZNS for LID were more apparent in the high-dose group (100 mg/kg). This may be explained by the different effects of different concentrations of ZNS in the striatum (Yamamura et al., 2009). Striatal perfusion of ZNS inhibited neurotransmission through the striato-external pallido GABAergic *indirect* pathway in a dose-dependent manner, enhancing LID. Striatal perfusion at higher concentrations increased dopamine levels in the striatum. Dopamine increases began at 40 min and reached the peak at 120 min, corresponding to the peak of AIMs in the high-dose group (100 mg/kg) in the present study (Figure 3). We should also mention that the effectiveness of high-dose ZNS on LID model mice could not be directly translated to clinical practice for PD patients although the metabolic rate is different between humans and mice.

The present study suggests that ZNS can improve LID by delaying its peak and reducing its severity before the peak although ZNS increases its duration. Additionally, ZNS may enhance the effects of l-DOPA and reduce its dosages (Murata et al., 2001, 2007, 2015; Murata, 2004), thereby prolonging the “honeymoon” period and delaying the appearance of LID. Further research should investigate the optimal dose of ZNS for clinical applications.

## Data availability statement

The original contributions presented in the study are included in the article/supplementary material, further inquiries can be directed to the corresponding author.

## Ethics statement

The animal study was reviewed and approved by the Institutional Animal Care and Use Committee of National Institutes of Natural Sciences.

## Author contributions

HS designed the project, performed the experiments, analyzed data, and wrote the manuscript. AN designed the project, wrote the manuscript, and helped in supervision. Both authors contributed to the article and approved the submitted version.
